# Utilization of zygotic embryos of an economic rattan palm *Calamus thwaitesii* Becc. (Arecaceae) for somaplant regeneration and cryobanking

**DOI:** 10.1007/s13205-012-0083-3

**Published:** 2012-08-07

**Authors:** A. S. Hemanthakumar, T. S. Preetha, P. N. Krishnan, S. Seeni

**Affiliations:** 1 Biotechnology and Bioinformatics Division, Tropical Botanic Garden and Research Institute, Palode, Thiruvananthapuram, 695 562 India; 2 Department of Botany, University College, Thiruvananthapuram, Kerala India

**Keywords:** *Calamus thwaitesii*, Cryopreservation, Embryo culture, Embryogenesis, Mass multiplication, Immature embryoids, Reintroduction

## Abstract

Zygotic embryos excised from immature green fruits of the rattan palm, *Calamus thwaitesii* and cultured for 16 weeks under optimum culture conditions in Murashige and Skoog (MS) medium supplemented with 31.67 μM 2,4-dichlorophenoxyacetic acid (2,4-D) and 35.23 μM 2,4,5-trichlorophenoxyacetic acid (2,4,5-T) produced mixed (compact and friable) calli at 70 and 92 % rates. The semi-friable part of the callus (~500 mg) separated and subcultured in medium containing 2.22 μM 6-benzyladenine and 1.07 μM α-naphthalene acetic acid produced groups of 10.37 ± 0.60–21.52 ± 0.48 discrete globular embryoids of varied size in 6–8 weeks. Calli raised in presence of 2,4,5-T were relatively more prolific, friable and embryogenic than those induced by 2,4-D. Embryoids (2.0–3.0 mm) isolated and cultured in basal medium germinated into plantlets at 65 % efficiency while the immature (0.5–2.0 mm) ones produced calloid structures. Approximately 15 % of the in vitro plantlets raised from the 2,4-D-induced embryogenic calli produced secondary immature embryoids on the sheath and lamina parts of leaves which were isolated and cultured in basal medium developed into rooted plantlets at 62 % rate in 12–16 weeks. The continued growth of the embryo-derived callus through successive subcultures together with differentiation of embryoids into plantlets, and the formation of immature embryoids on in vitro plantlets in MS basal nutrient medium reports for the first time a reliable method of producing at least 116 plants from a single embryo in a year. Rooted plantlets treated with 50 % glycerin survived at 78 % rate after hardening and 82.7 % of the hardened plants reintroduced into forest segments showed uniform growth free of morphological abnormalities after 3 years of observation. In addition to embryogenesis, cryopreservation of the zygotic embryos through simple drying and encapsulation–dehydration methods resulting 60–70 % recovery rates also offers another option for long-term conservation and sustainable utilization of this plant genetic resource.

## Introduction

Rattans are unique and versatile group of spiny climbing palms or canes with solid stem distributed in the forests of Indo-Malayan and African regions. In India they are represented by 5 genera and 51 species occurring in the tropical evergreen, semi-evergreen and moist deciduous forests of the Western Ghats, Eastern Himalayas and Andaman Islands. Species of *Calamus* distributed in the Western Ghats region of southern peninsula are a high value non-timber forest product primarily used for making furniture, baskets and handicraft items and an important raw material of the cottage industry contributing employment to approximately 1,00,000 people in the state of Kerala alone (Bhat et al. 1989). In the post-independent period, factors such as injudicious exploitation, unscientific harvesting, habitat degradation and plantation activities in the hills have resulted in serious depletion of rattan genetic resources in the region, poor regeneration in natural forests and consequently increased gap between demand and supply (Rao et al. 1990). Due to shrinkage of wild rattan resources and non-availability of raw materials, the rural industrial units are either closed down or forced to receive their supplies from far away Assam and Andaman islands, causing significant increase in the prices of cane products and jeopardizing the livelihood of workers involved in the local extraction and processing of canes. The most seriously affected economically important species in Kerala State in India is *Calamus thwaitesii* which despite its wide distribution is indiscriminately exploited to the extent that mature useful canes are no more available in the accessible forests. Species of *Calamus* are dioecious, seed set is poor and seed availability in the disturbed habitats is uncertain. Besides, extraction of canes is often done before flowering and hence seed bearing mature palms are confined to protected forests in wildlife sanctuaries and national parks.

In cases where seed availability is scarce or traditional propagation methods are impractical, artificial regeneration including micropropagation holds promise for the large-scale production of planting materials (Wochok 1981). Micropropagation of Indian rattans is not worked out (Singh et al. 2004) though there are isolated reports on single seedling formation in embryo cultures of *C. rotang* and multiple shoot initiation in embryo callus of *C. gamblei* (Padmanabhan and Ilangovan 1989, 1993). In this paper, we report for the first time embryogenesis through embryo callus cultures contributing to conservation, significant multiplication and commercial utilization of *C. thwaitesii* by re-establishment of the plants so multiplied in native forest segments. Besides, due to recalcitrant nature of seeds, zygotic embryo cryopreservation was also carried out to ensure the long-term in vitro conservation of this commercially exploited non-wood forest produce.

## Materials and methods

### Plant materials

Bunches of unripe, green fruits of *C. thwaitesii* were collected in the month of November from selected elite palms growing in the Herbal Garden of the Kerala State Forest Department at Arippa in Kollam District of Kerala. The fruits separated from the rachis were thoroughly washed in running tap water for 3–5 min before being treated with 0.8 % (v/v) Teepol (BDH India Ltd, Bombay) for 10 min and rinsed several times in distilled water. Surface decontamination of the fruits was done by immersion in 70 % ethanol and flaming inside the laminar air flow hood. Embryos were dissected out of the fruits and washed once in sterile distilled water before transferred to the nutrient medium.

### Initiation of embryogenic callus cultures

To study the effect of auxins on embryogenic callus formation, the immature zygotic embryos were cultured on full strength MS medium (Murashige and Skoog 1962) pH 5.8; 0.6 % agar (SRL Laboratory, Pvt. Ltd., Mumbai) supplemented with 4.52–54.29 μM 2,4-D or 3.91–46.97 μM 2,4,5-T. Each treatment consisted of 25 embryos with three replicates. All the embryos with or without callus were transferred to fresh medium of the same composition after 8 weeks of culture. The cultures were incubated at 24 ± 2 °C under diffused light (1–5 μmol m^−2^ S^−2^).

### Induction of embryoids, plantlet regeneration and cryopreservation

The white semi-friable calli obtained from the embryos after 16 weeks of culture were divided into ~500 mg pieces and subcultured to medium supplemented with varied combinations of BA (0.89, 2.22, 4.44 μM) and NAA (0.54, 1.07, 2.69 μM) to induce differentiation of embryoids. After 8 weeks of subculture, 2.0–3.0 mm size embryoids differentiated upon the callus were separated and cultured in the basal medium for 8–16 weeks to induce plantlet development. The callus with the remaining young embryoids were subcultured in basal medium through 2–3 cycles of 8 weeks each to facilitate maturation of the embryoids and from them plantlet formation. So also the somatic embryoids formed on the sheath and lamina parts of the leaves were isolated and cultured again in basal medium for 8–16 weeks to obtain rooted plantlets.

Embryoids liberated from the callus mass were subjected to free-hand sectioning, stained with few drops of 2 % aqueous toluidine blue and examined under Nikon SMZ 800 stereomicroscope. As an alternative strategy for long-term conservation, cryopreservation of zygotic embryos through simple drying (desiccation inside the laminar air flow for 30 min to 8 h) and encapsulation–dehydration (Fabre and Dereuddre 1990) methods were carried out.

### Hardening and field establishment

The plantlets (each with 2–3 leaves and 1–3 roots) weaned away from the flasks were washed repeatedly in running water and treated with 0.1 % Dithane M45 (Indofil Chemical Company, Mumbai) to avoid fungal infection. They were then transplanted in polybags filled with a mixture of river sand, top soil and farm yard manure (3:1:1), irrigated and hardened in a mist chamber (80 ± 5 % RH; 28 ± 2 °C) specially fabricated for the purpose by Indo-American Exports Ltd., Bangalore. If necessary, the plantlets were dipped in 50 % glycerin before hardening to prevent evapotranspiration. After 3–4 weeks of hardening, the polybag plantlets were transferred to an agro-shade house and reared under 50 % sunlight and regular watering. After 3 months, all the established palms were planted into two forest segments of the institute campus at 5 m spacing during the south-west monsoon (June–July) season of 2006 and monitored at monthly intervals for their establishment and growth through 3 years.

### Experimental design, data collection, and statistical analysis

For embryogenic callus formation, each culture flask (250 ml) was inoculated with 5 embryos, with 25 replicates used for each treatment. Responses of embryos were recorded as a percentage based on the number of embryos showing germination/organ formation, callusing and browning/necrosis after 16 weeks of inoculation. Data on embryoid formation from embryogenic calli after 8 weeks of culture and percentage of establishment, mean number of new leaves after 3 years of reintroduction was recorded. Besides, the data on recovery of cryopreserved zygotic embryos were also collected after 5 weeks of rewarming. All the experiments were repeated at least three times. Data was subjected to analysis of variance (ANOVA) using a completely random design (CRB) and means were compared by Duncan’s multiple range tests.

## Results

**Fig. 1:**
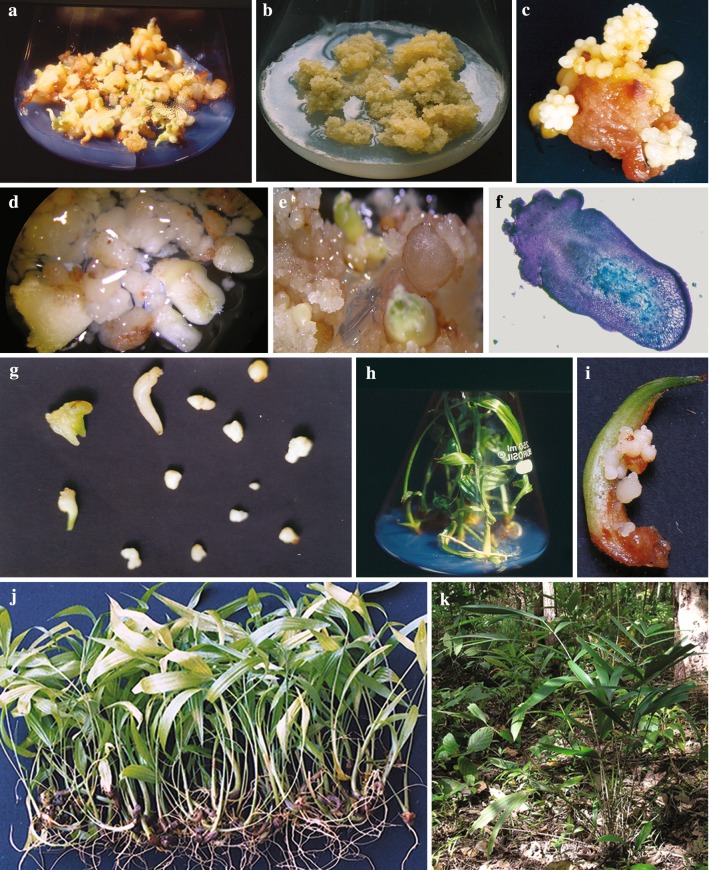
In vitro embryogenesis in *Calamus thwaitesii.***a** High 2,4-D (>31.67 μM) induced embryo callus forming shoots after 12 weeks of culture in MS medium. **b** Semi-friable to amorphous calli formed during subculture in 35.23 μM 2,4,5-T. **c** Clusters of embryoids differentiated upon 2,4-D-induced embryo callus cultured in 2.22 μM BA and 1.07 μM NAA. **d**, **e** Embryogenic callus showing the formation of loose off-white to light green embryoids and conversion of embryoid into shoot after 8 weeks of culture. **f** LS of isolated embryoids showing shoot primordium and independent vasculature. **g** Germination of embryoids in MS basal medium after 4–8 weeks of culture. **h** Rooted plantlets raised from embryoids after 16 weeks. **i** Secondary immature embryoids formed on leaf sheath and lamina of plants raised from 2,4-D (31.67 μM) induced embryogenic callus. **j** Deflasked plantlets ready for hardening. **k** Three-year-old micropropagated plant established in the forest floor

Invariably all the embryos cultured in MS basal medium under diffuse light swelled 3–4 times their original size followed by the formation of root and shoot poles along with haustorial enlargement in 3 weeks and development of seedlings each with 2.0–4.0 cm shoot and 1–3 roots of varied length in 8–12 weeks. Supplementation of the medium with varied concentrations of 2,4-D/2,4,5-T was required to reverse the organized development of embryos (Table 1). Swelling of the embryos observed within 4–6 weeks in media fortified with 9.05 μM or less of 2,4-D was accompanied by the emergence of normal shoot and root. The percentage and degree of callusing induced by 2,4,5-T at the concentrations tested was substantial. While callusing followed by differentiation of shoots and roots was observed in 5–15 % of the embryos cultured even in high concentrations (31.67 μM) of 2,4-D (Fig. 1a), organogenesis was altogether arrested at concentrations exceeding 11.74 μM 2,4,5-T (Table 1). The calli proliferated upon the embryos contained both compact, and semi-friable portions, the friability increasing with increasing concentrations of the auxins. The pronounced compact and translucent callus formation observed in presence of 2,4-D was absent in medium containing 27.4–39.14 μM 2,4,5-T; however, no particular type of callus was uniformly produced in both the types and concentrations of the auxins tested. After 16 weeks of culture, the optimum concentration required for maximum percentage of semi-friable callus formation and extent of semi-friable callusing in each embryo varied between the two auxins with 70 % of the embryos producing white, semi-friable growing callus at 31.67 μM 2,4-D and 92 % of the embryos responding with white, semi-friable to amorphous rapidly growing callus formation at 35.23 μM 2,4,5-T. About 8–12 % of the embryos cultured in presence of these auxins did not respond at all to callus formation but changed their colour to yellowish brown or brown during the 16-week culture period. Though increasingly semi-friable calli, formed at concentrations exceeding the optimum showed signs of browning and at 45.24 μM 2,4-D and 46.97 μM 2,4,5-T; 15–32 % of the embryos were lost due to browning and necrosis.

**Table 1:** Response of *Calamus thwaitesii* zygotic embryos cultured in MS medium supplemented with varied concentrations of 2,4-D and 2,4,5-T

Plant growth regulator (μM)	Germination/organ formation (%)	Callusing (%)	Browning/necrosis (%)
2,4-D			
00.00	100.0 ± 0.00^a^	00.00 ± 0.00	00.00 ± 0.00
04.52	97.33 ± 1.33^a^	00.00 ± 0.00	2.67 ± 1.33^j^
09.05	74.67 ± 1.33^c^	20.00 ± 2.31^hi^	5.33 ± 3.53^j^
13.57	56.00 ± 2.31^e^	41.33 ± 1.33^f^	2.67 ± 1.33^j^
18.10	46.67 ± 1.33^f^	50.67 ± 1.33^ef^	2.67 ± 1.33^j^
22.62	41.33 ± 1.33^f^	54.67 ± 2.67^e^	4.00 ± 2.31^j^
27.14	37.33 ± 1.33^g^	60.00 ± 2.31^de^	2.67 ± 1.33^j^
31.67	22.67 ± 3.53^h^	70.67 ± 1.33^cd^	6.67 ± 4.81^j^
36.19	21.33 ± 3.53^hi^	69.33 ± 1.33^d^	9.33 ± 4.81^ij^
40.72	20.00 ± 4.62^hi^	68.00 ± 2.31^d^	12.0 ± 6.93^ij^
45.24	10.67 ± 1.33^ij^	66.67 ± 1.33^d^	22.67 ± 2.67^h^
49.76	09.33 ± 1.33^ij^	62.67 ± 1.33^d^	28.00 ± 2.31^h^
54.29	06.67 ± 1.33^j^	60.00 ± 2.31^de^	33.33 ± 1.33^g^
2,4,5-T			
03.91	50.67 ± 1.33^e^	45.33 ± 3.53^f^	04.00 ± 2.31^j^
07.83	30.67 ± 1.33^g^	66.67 ± 1.33^d^	02.69 ± 1.33^j^
11.74	00.00 ± 0.00	80.00 ± 4.62^bc^	20.00 ± 4.62^hi^
15.66	00.00 ± 0.00	81.33 ± 1.33^b^	18.67 ± 1.33^i^
19.57	00.00 ± 0.00	82.67 ± 1.33^b^	17.33 ± 1.33^i^
23.48	00.00 ± 0.00	85.33 ± 2.67^b^	14.67 ± 2.67^i^
27.40	00.00 ± 0.00	89.33 ± 1.33^b^	10.67 ± 1.33^i^
31.31	00.00 ± 0.00	90.67 ± 2.67^ab^	09.33 ± 2.67^ij^
35.23	00.00 ± 0.00	93.33 ± 1.33^a^	06.67 ± 1.33^j^
39.14	00.00 ± 0.00	84.00 ± 2.31^b^	16.00 ± 2.31^i^
43.06	00.00 ± 0.00	81.33 ± 2.67^bc^	18.67 ± 2.67^i^
46.97	00.00 ± 0.00	80.00 ± 2.31^bc^	20.00 ± 2.31^hi^

Data represents mean ± SE of 25 replicates repeated thrice, recorded after 16 weeks of culture through two passages of 8 weeks each. Means followed by the same superscript do not differ significantly at 5 % level based on ANOVA and Duncan’s multiple range tests

**Fig. 2:**
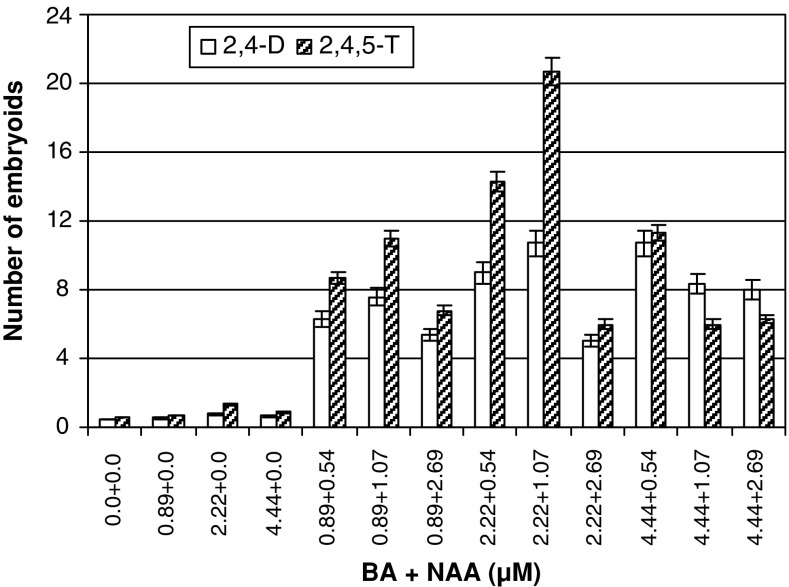
Embryoid formation from embryogenic calli developed from 2,4-D or 2,4,5-T treatment on MS medium supplemented with varied concentrations of BA and NAA. Observations were made after 8 weeks of culture

The semi-friable portion dissected out of the embryo callus and subcultured for 8 weeks in the medium containing 31.67 μM 2,4-D or 35.23 μM 2,4,5-T continued to proliferate into increasingly semi-friable callus or amorphous callus in the latter as the case may be (Fig. 1b). However, subsequent division of the callus so formed into 3–4 pieces of 500 mg fresh weight and subculturing of each callus piece in media fortified with BA and NAA showed only marginal growth of the callus but differentiation into 10.37 ± 0.60 to 21.52 ± 0.48 white coloured embryoids occurred in 6–8 weeks particularly in a combination of 2.22 μM BA and 1.07 μM NAA. Though embryoids varying in size, shape and stage of development were recorded in calli derived from both the auxin types, 2,4,5-T induced calli formed significantly higher number of embryoids than 2,4-D-induced one, especially in presence of 2.22 μM BA and 1.07 μM NAA (Fig. 2). Besides, the 2,4-D-induced embryoids remained in clusters (Fig. 1c) while those induced by 2,4,5-T were discrete off-white loose structures some of which turned light green and were easily separated (Fig. 1d, e). A few chlorophyllous embryoids so formed germinated into shoots on the callus. Free-hand sections of the isolated embryoids revealed the presence of independent vasculature and organized shoot primordium (Fig. 1f). On culture in MS basal medium, majority (65 %) of the mature embryoids (2.0–3.0 mm) developed shoots and roots in 4–8 weeks (Fig. 1g) and well-developed plantlets in 12–16 weeks (Fig. 1h). The younger embryoids (0.5–1.0 mm) devoid of distinct shoot pole invariably produced calloid structures deficient in morphogenetic response.

Passage of the 2,4-D-induced callus with the embryoids through two subculture cycles of 8 weeks each in basal medium permitted the germination of most of the embryoids and emergence of shoot and root leading to plantlet formation. However, differentiation of new embryoids continued from the growing portion of the embryogenic callus. While the older portion of the callus ceased to grow and turned yellowish brown, the growing callus was somewhat compact, off-white and the undifferentiated embryoids white in colour. Surgical removal and subculture of the growing embryogenic callus through 1–2 cycles of 8 weeks each in media supplemented with 2.22 μM BA and 1.07 μM NAA produced embryoids of various developmental stages which were separated and transferred to the MS basal medium at 12–16 week intervals to increase the number of plantlet formation. In fact the embryogenic potential of 2,4-D-derived cultures was so high that secondary immature embryoids were formed on the leaf sheath and lamina portion of about 15 % of the regenerated plantlets (Fig. 1i). Plantlets raised from 2,4,5-T induced embryogenic callus cultures were free from immature embryoid formation. The immature embryoids separated and cultured in basal nutrient medium for 8–16 weeks developed into plantlets at 62 % success rate. Altogether, a maximum of 116 rooted plantlets were produced from the culture of a single zygotic embryo in a year.

Difficulties were encountered during establishment of the deflasked plants in polybags. In spite of the well-developed root system of the plantlets and hardening procedure employed, more than 60 % mortality was recorded within 8–12 weeks in the initial trials. However, 78 % establishment was achieved when the plantlets were treated with 50 % glycerin before hardening (Fig. 1j). Twelve weeks after hardening, the plants reintroduced into forest segments of the institute campus before the south-west monsoon rains showed signs of growth with the emergence of a new leaf in 8 weeks. The plants established at 82.7 % rate showed uniform growth and were free from morphological and growth abnormalities during the 3 years of observation in the field (Table 2). After 3 years of establishment, each in vitro-derived palm had already formed 5–7 leaves with spines all over the body (Fig. 1k).

**Table 2:** Reintroduction of embryogenesis-derived plants of *C. thwaitesii* into selected forest segments of TBGRI campus

Forest segments	Total no. of plants introduced	Plants established	Percentage establishment	Mean no. of new leaves
1	150	124	82.7	6.42 ± 0.16
2	260	205	78.8	6.33 ± 0.21

Three-month-old hardened plants were reintroduced into the forest segments during May–June 2006 before the onset of south-west monsoon. Data on the establishment of the plants were collected after 3 years in June 2009

**Fig. 3:**
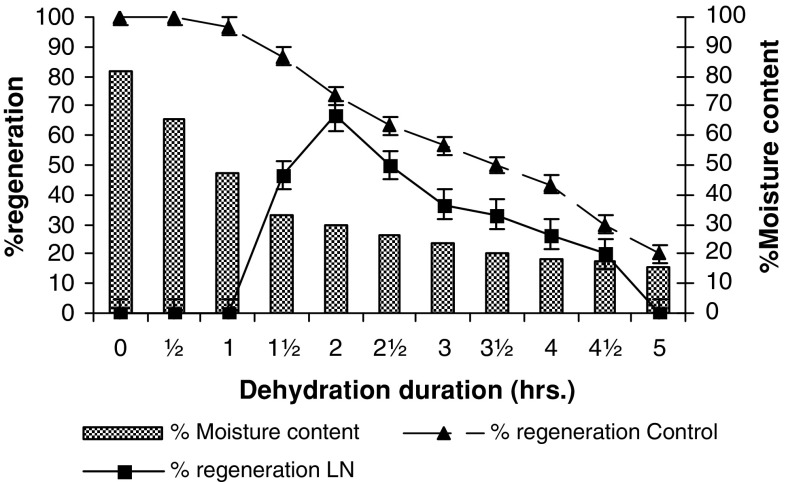
Recovery of cryopreserved zygotic embryos in simple drying method. Values are mean ± SE of 10 replicates repeated thrice. Observations were made after 5 weeks of rewarming

**Fig. 4:**
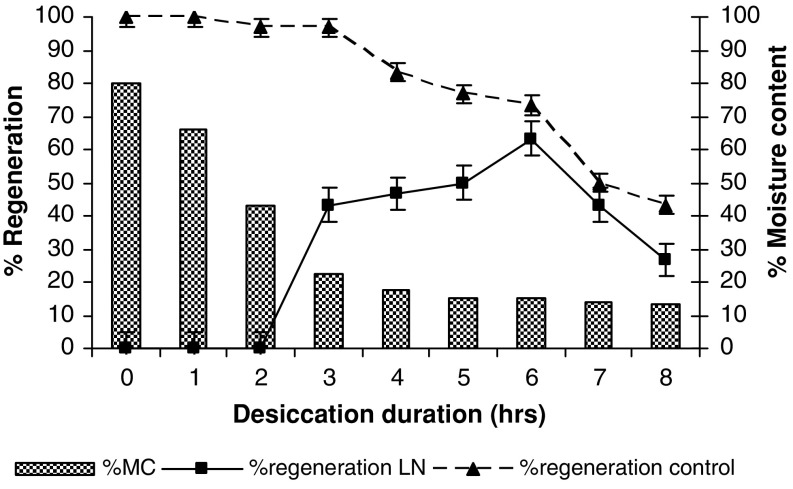
Recovery of cryopreserved zygotic embryos using encapsulation–dehydration method. Values are mean ± SE of 10 replicates repeated thrice. Observations were made after 5 weeks of rewarming

Zygotic embryos were successfully cryopreserved using simple drying and encapsulation–dehydration methods. In simple drying method, embryos desiccated for 2 h with a moisture content of 29.69 % exhibited 66.67 % regeneration after cryopreservation (Fig. 3). In encapsulation–dehydration method, embryos encapsulated in calcium alginate matrix after preculture in 0.5 M sucrose osmotically dehydrated for 6 h with 14.98 % moisture content also responded with 63.33 % regeneration in cryopreserved embryos (Fig. 4).

## Discussion

In order to augment planting material production and supply in rattan palms, embryos as opposed to vegetative tissues of mature palms are the choice explants for in vitro culture of both clustering (Dekkers and Rao 1989; Padmanabhan and Ilangovan 1993) and solitary (Yusoff and Manokaran 1985; Yusoff 1989) types. With a few exceptions, the embryos were released from the mature fruits after cut open the hard endosperm and cultured invariably in MS medium to produce single seedling (Padmanabhan and Ilangovan 1989; Chuthamas et al. 1989) or callus in which caulogenesis was induced later (Gunawan and Yani 1986; Yusoff 1988; Padmanabhan and Ilangovan 1993). In the present study, the use of somewhat immature green fruits of *C. thwaitesii* collected in the month of November was found most suitable to release with ease the embryos more than 95 % of which were free from damage. The endosperm of the ripe fruits collected in the month of February being stony, were broken with difficulty to yield embryos of which >70 % were damaged and could not be used for culture. Nutrient formulation of MS without supplementation was sufficient to induce germination of all the immature embryos into typical seedlings with normal haustorium, root and shoot formations as in mature embryos (Padmanabhan and Ilangovan 1989) while auxin supplementation promoted callus proliferation. Therefore, we recommend the use of relatively younger fruits for easy isolation and culture of intact embryos in rattan palms.

In both embryo and tissue cultures of rattan palms (Dekkers and Rao 1989; Yusoff 1989; Padmanabhan and Ilangovan 1993; Kundu and Sett 1999; Sett et al. 2002), date palm (Reynolds and Murashige 1979), coconut (Gupta et al. 1984) and oil palm (de Touchet et al. 1991; Teixeira et al. 1994), 2,4-D was the most frequently used auxin to produce calli which were subsequently used for plant regeneration through indirect organogenesis. Though the concentration (31.67 μM) of 2,4-D optimized for callus formation in embryo culture of *C. thwaitesii* is in broad consensus with the concentrations used by other workers in different *Calamus* species, the results obtained in the present study suggest that 2,4,5-T is superior to 2,4-D in inducing consistent formation of semi-friable and embryogenic calli in 92 % of the embryos. The normal development of the excised embryo to produce shoot and roots had to be reversed and this was better achieved with 2,4,5-T at lower concentration (11.74 μM) than with 2,4-D at high (31.67–45.24 μM) concentrations. Consequently, even at the suggested optimal concentration of 31.67 μM 2,4-D, 20 % of the embryos still formed roots and shoots with calloid outgrowth. In addition to the tendency to form organs to varied extent through the entire range of concentrations tested, the 2,4-D-induced calli differed significantly in morphology, texture and physiology. After 16 weeks of culture, the compact and translucent portion occupied significant portions of the callus, and the subcultured semi-friable portion produced relatively few (10.37 ± 0.60) embryoids in medium containing BA and NAA. On the contrary, the 2,4,5-T induced calli were essentially semi-friable, tend to be amorphous during subculture and very much embryogenic with the formation of large number (21.52 ± 0.48) of embryoids as discrete loose structures that could be easily separated. Although somewhat compact type callus was recorded in low concentrations of this auxin, the translucent callus was never formed in any of the concentrations tested. These results together with the unpublished data obtained in embryo cultures of other species of *calamus* (*C. rotang, C. hookerianus, C. travancoricus* and *C. nagabettai*) suggest that 2,4,5-T is a stronger synthetic auxin than 2,4-D to induce increasingly friable, amorphous and embryogenic callus formation. The amorphous callus with the loosely held embryoids formed during subculturing of the essentially semi-friable callus may indicate the utility of such a callus for establishing suspension cultures of embryoids to achieve mass multiplication of the species. This is not possible with the 2,4-D-induced semi-friable callus which seldom became amorphous. Both the auxins proved to be toxic at concentrations exceeding 54.29 μM for 2,4-D and 46.97 μM for 2,4,5-T, resulting in 15–30 % loss of the embryos due to browning and necrosis. The loss of 8–12 % of the non-responding embryos due to eventual yellowing or browning during culture in presence of the optimal concentrations of the auxins, is a matter for further investigation as possible irreversible damage of the embryos occurring during isolation could not be ruled out. The diversity of responses, viz. organogenesis, callusing and browning observed in the embryos subjected to a given treatment indicated their physiological heterogeneity.

In most of the published works on rattans, the embryos or tissues were first cultured in a high auxin medium to produce callus and then the callus subcultured in medium containing high cytokinin–auxin ratio to induce multiple shoot formation. This invariably involves a number of subcultures of embryo callus before organogenetic competence could be induced as in *C. manan* (Rao et al. 1990). The shoots were then separated and rooted to obtain complete plantlets. The rapid multiplication achieved in the present study with initial callusing of embryos on media containing different concentrations of 2,4-D and 2,4,5-T in 16 weeks followed by division of the callus into ~500 mg pieces, subculture in medium amended with BA and NAA to produce 10.37 ± 0.60–21.52 ± 0.48 embryoids in 6–8 weeks and germination of mature embryoids into plantlets in basal nutrient media is qualitatively different and confirms the higher propagation potential of *C. thwaitesii* through the embryogenic rather than organogenetic route. It is particularly so with calli raised with 2,4,5-T which induced enhanced friability and embryogenic potential of the callus tissues. In fact, the demonstrated ability to produce up to 116 plantlets from a single zygotic embryo within a year in *C. thwaitesii* confirms the desirability of using embryogenic rather than indirect organogenetic route otherwise reported in general, and in *C. flagellum* (Kundu and Sett 1999) and *C. tenuis* (Sett et al. 2002) in particular. The proliferation of embryogenic callus in presence of 2,4-D or 2,4,5-T, concomitant formation of embryoids from the callus in medium supplemented with low concentrations of BA and NAA, repeated harvest of embryoids through 2–3 subculture cycles and development of embryoids into plantlets in basal medium altogether form a novel plant regeneration system unrecorded earlier in *Calamus* species. The embryogenic competence of the tissues was high so that 15 % of the plants raised from the 2,4-D-induced embryogenic calli formed secondary immature embryoids on the petiole and lamina parts which were separated and cultured in basal medium to germinate into individual plants at 62 % success rate. The mature embryoids (2.0–3.0 mm) derived from the zygotic embryo callus, being organized bipolar structures, on culture in basal nutrient medium individually or as part of the callus were converted into rooted plantlets at 65 % efficiency. The immature embryoids were callogenic and were probably not differentiated or determined to the extent of developing into mature embryos or plantlets as the case may be. The results suggest that further refinement is particularly needed to convert all the embryoids obtained from different sources into plantlets.

The formation of secondary immature embryoids in clusters on leaf sheath and lamina of plants raised from embryoid-bearing calli served as an additional source of regeneration and multiplication of *C. thwaitesii*. There is as yet perhaps no precedence of somatic embryogenesis on in vitro-raised rattan plants. In date palm, however, Sudhersan et al. (1993) reported the formation of somatic embryos on the young leaf lamina of in vitro plants. Since production of embryoids ceases after 2–3 subcultures of the embryo callus, it would be worth investigating the establishment of embryogenic suspension cultures from the zygotic amorphous embryo callus obtained during subculture in presence of 2,4,5-T or from the proembryogenic cell masses proliferated on the leaves of in vitro plants for continuous production of embryoids.

In the initial trials, more than 60 % of the rooted plantlets were lost during the hardening and post-hardening phases in the nursery. The method of coating of the plantlets with 50 % glycerin originally developed for other species Selvapandyan et al. (1988) helped to achieve 78 % establishment thereby indicating lack of epicuticular wax formation and possibly ineffective stomata on the leaves of in vitro-derived plants due to high humidity and also by low levels of illumination (Sutter and Langhans 1982) during the culture period. Perhaps the dark respiration of the micropropagated plants as suggested by Yusoff (1988) may also play certain role in plant establishment after weaning. Finally, the high rate of survival (82.7 %) and continued performance of the plants in the forests without morphological and growth abnormalities and formation of 5–7 new leaves and spines all over the plant body during the 3 years after planting confirmed the uniformity of the plants.

From conservation point of view, cryopreservation holds great promise for long-term storage, consistent production and sustainable utilization of the plant genetic resources. Development of effective cryopreservation protocols for tropical plants producing recalcitrant seeds finds wide acceptance as they are not exposed to cold or freezing temperature or extreme desiccation during their normal life cycle (Natarajan et al. 2008). Cryopreservation of zygotic embryos of this recalcitrant species was successfully done using simple drying or encapsulation dehydration method showing 60–70 % regeneration in cryopreserved embryos desiccated at 15–30 % moisture content. Moisture content between 15 and 25 % is required for optimal survival after cryopreservation (Engelmann 1992) which was also the case in their study. Among the two methods tested, simple drying is easy to perform compared to encapsulation dehydration method. Usually preculture in high sucrose followed by dehydration is used for cryopreserving zygotic embryos of other palm species (Assy-Bah and Engelmann 1992). In contrast, we have achieved successful cryopreservation of embryos of *C. thwaitesii* using simple dehydration method without sucrose preculture. All these results suggest that embryogenesis in zygotic embryo callus cultures and cryopreservation is yet another option for conservation, production and supply of high-quality planting materials of rattan palms.

## Abbreviations

BA: 6-Benzyladenine
MS: Murashige and Skoog
2,4-D: 2,4-Dichlorophenoxyacetic acid
2,4,5-T: 2,4,5-Trichlorophenoxyacetic acid
NAA: α-Naphthaleneacetic acid
PGR: Plant growth regulator
LN: Liquid nitrogen
